# Implementing sequence-based antigenic distance calculation into immunological shape space model

**DOI:** 10.1186/s12859-020-03594-3

**Published:** 2020-06-19

**Authors:** Christopher S. Anderson, Mark Y. Sangster, Hongmei Yang, Thomas J. Mariani, Sidhartha Chaudhury, David J. Topham

**Affiliations:** 1 Department of Pediatrics, University of Rochester Medical Center, University of Rochester School of Medicine and Dentistry, Rochester, NY USA; 2 New York Influenza Center of Excellence at David Smith Center for Vaccine Biology and Immunology, Department of Microbiology and Immunology, University of Rochester School of Medicine and Dentistry, Rochester, NY USA; 3 Department of Biostatistics and Computational Biology, University of Rochester Medical Center, Rochester, NY USA; 4 Center for Enabling Capabilities, Walter Reed Army Institute of Research, Silver Spring, MD USA

**Keywords:** Gillespie algorithm, Shape space, Antigenic distance, Epitopes, Antigenic sites, Hemagglutinin, Influenza, Vaccines, Computational immunology, HA, Stalk, Stem, 2009 pandemic, H1N1, pH1N1, Artificial immune systems, Humoral immune system, Simulations

## Abstract

**Background:**

In 2009, a novel influenza vaccine was distributed worldwide to combat the H1N1 influenza “swine flu” pandemic. However, antibodies induced by the vaccine display differences in their specificity and cross-reactivity dependent on pre-existing immunity. Here, we present a computational model that can capture the effect of pre-existing immunity on influenza vaccine responses. The model predicts the region of the virus hemagglutinin (HA) protein targeted by antibodies after vaccination as well as the level of cross-reactivity induced by the vaccine. We tested our model by simulating a scenario similar to the 2009 pandemic vaccine and compared the results to antibody binding data obtained from human subjects vaccinated with the monovalent 2009 H1N1 influenza vaccine.

**Results:**

We found that both specificity and cross-reactivity of the antibodies induced by the 2009 H1N1 influenza HA protein were affected by the viral strain the individual was originally exposed. Specifically, the level of antigenic relatedness between the original exposure HA antigen and the 2009 HA protein affected antigenic-site immunodominance. Moreover, antibody cross-reactivity was increased when the individual’s pre-existing immunity was specific to an HA protein antigenically distinct from the 2009 pandemic strain. Comparison of simulation data with antibody binding data from human serum samples demonstrated qualitative and quantitative similarities between the model and real-life immune responses to the 2009 vaccine.

**Conclusion:**

We provide a novel method to evaluate expected outcomes in antibody specificity and cross-reactivity after influenza vaccination in individuals with different influenza HA antigen exposure histories. The model produced similar outcomes as what has been previously reported in humans after receiving the 2009 influenza pandemic vaccine. Our results suggest that differences in cross-reactivity after influenza vaccination should be expected in individuals with different exposure histories.

## Background

For rapidly antigenically-drifting viruses, such as the influenza virus, the amount of long-term protection provided by an antibody depends partly on where precisely on the virus the antibody binds. Upon first exposure to influenza virus (or influenza-derived viral antigens), randomly-assembled immunoglobulin receptors on naïve B cells will bind specific parts of the virus and form germinal centers, resulting in differentiation of long-lived memory B cells and antibody-secreting cells specific to that part of the virus [1]. Upon a second exposure to an antigenically-drifted influenza virus, some memory B cells (and antibodies) will lose their affinity for the virus if enough change has occurred in the specific part of the virus the antibody binds [2–6]. Influenza vaccines aim to induce antibody to the attachment protein hemagglutinin (HA). HA is typically divided into five, non-overlapping, regions that are known to elicit an antibody response (i.e. antigenic-sites). Although the HA protein rapidly undergoes antigenic drift to avoid these antibodies, some specific parts of the influenza virus, such as the “stalk” region of the HA protein, undergo a less rapidly antigenic drift [5, 7, 8]. Therefore, memory B cells with immunoglobulin receptors specific to these more conserved parts of the virus will still be able to bind (i.e. be cross-reactive to the antigenically drifted virus) and will quickly form germinal centers resulting in additional memory B cells and antibody-secreting cells specific to the conserved part on the virus [7, 9, 10].

In 2009, an antigenically distinct strain of influenza H1N1 virus jumped from swine to humans and caused a world-wide pandemic [11]. During the pandemic, a vaccine containing the HA protein of the 2009 H1N1 pandemic strain was distributed to the population. Studies of the resulting antibody responses to the 2009 vaccine demonstrated that the level of antibody cross-reactivity, the number of distinct antigens an antibody can bind, differed depending on age, with younger-age individuals, those not exposed to early twentieth century viruses, showing greater cross-reactivity compared to older-aged individuals [70+ years old] who were likely exposed to twentieth century viruses at a young age [7, 11, 12]. Younger-aged individuals in 2009 showed increased levels of antibodies towards the highly-conserved stalk region of HA, while older-aged individuals showed a typical response, mounting antibodies predominantly towards parts of the head of HA [7, 10, 12, 13]. Further studies showed that these differences in antibody specificity after vaccination with the 2009 vaccine were due to variations in the levels of pre-existing antibody and memory B cells cross-reactive to the 2009 pandemic influenza virus; older-aged individuals having been exposed to early twentieth century influenza viruses that were antigenically similar to the 2009 pandemic virus while younger-aged individuals had only been exposed to the recently circulating antigenically distinct influenza strains [14].

Representing real-life influenza virus HA antigens in a model requires estimation of the antigenic distance (AD) between the HA antigens of antigenically-distinct influenzas viruses. AD is the property of two antigens where the shorter antigenic distance between antigens the greater number of antibodies that will be able to bind both antigens. Many methods to determine the AD between HA antigens have been developed and applied [3, 4, 9–19]. We recently developed a computational algorithm (SBM.v1) for determining the AD between H1N1 HA antigens using publicly available influenza genome sequences [4]. Importantly, the SBM.v1 method estimates the antigenic distances for individual parts of the virus (antigenic-sites) across the HA antigen [4, 5].

Many computation models have been developed to model immune responses to influenza virus [19–22]. Most of these models do not explicitly model where on the virus the antibody binds and therefore do not capture the increased antibody cross-reactivity expected when antibodies targeting more conserved regions dominate the immune response. Here we estimate the antigenic-site-specific antigenic distances between HA antigens from historical H1N1 strains using the SBM.v1 method [4]. We introduce the ssMod.v1 which explicitly represents agents in the humoral immune system. ssMod.v1 allows explicit representation of 5 canonical H1N1 HA antigenic-sites and a conserved HA-stalk antigenic-site [23]. We performed computer simulations representing individuals from different age-epochs known to have differences in specificity of pre-existing immunity. We simulated humoral immune responses to the 2009 H1N1 monovalent vaccine and output the predicted antibody specificity and cross-reactivity to a set of antigenically distinct HA antigens during the simulation. Our goal is to generate a model that will correctly predict the differences in antibody specificity and cross-reactivity seen after vaccination with the 2009 pandemic vaccine resulting from differences in pre-existing immunity. We compare the model results with human serum antibody levels obtained from a small clinical trial and discuss the results in regards to other published studies.

## Results

### Estimation of AD between HA antigens

**Fig. 1:**
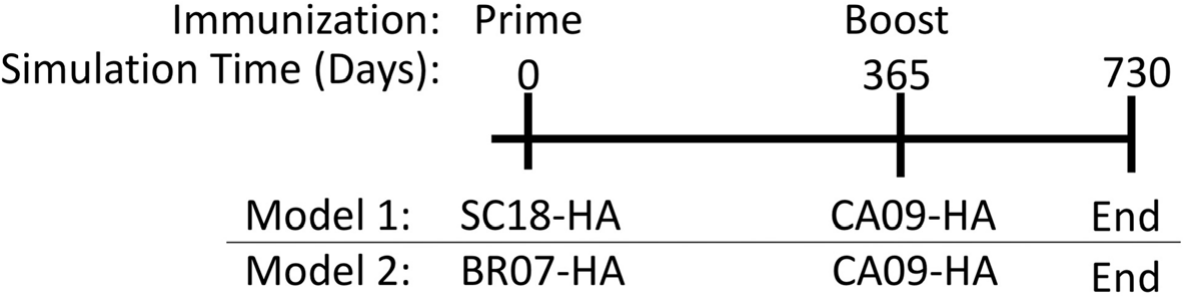
Immunization Strategy Diagram. Two models were constructed. In one model, exposure to SC18 HA antigen (prime) was simulated at day 0 and exposure to CA09 HA antigen (boost) was simulated 365 days later. The second model was identical to the first except that BR07 HA antigen was added at day 0. Simulations were carried out for a total of 730 days. B cell and antibody specificities, genotypes, and numbers were tracked throughout the simulation

AD between HA antigens represented in the model was determined using the SBM.v1 algorithm [4]. The ADs between 11 antigenically distinct H1N1 HA antigens were determined. Three strains were used as immunogens (viral HA antigen) in the model (Fig. 1) including the 1918-pandemic strain, A/South Carolina/1/1918 (SC18); the 2007 vaccine strain, A/Brisbane/59/2007 (BR07); and the 2009-pandemic vaccine strain, A/California/07/2009 (CA09). The other strains included in the model (see Methods) were used to assess the cross-reactivity of the antibodies during the simulation.

HA antigens for each strain was represented in the model as six, 20-character, strings representing 6 antigenic-sites. These 6 antigenic-sites represent the 5 canonical head antigenic-sites (Sa, Sb, Ca1, Ca2, Cb) [24] and 1 stalk antigenic-site (Stk) [12]. Strings were created in such a way that the Hamming distance between them was equivalent to the antigenic distance estimated for each of the 5 antigenic-sites on the head region of HA. The antigenic-site representing the stalk region of HA was kept fully conserved between all HA antigens (AD = 0).

In the model, SC18 and CA09 HA antigens were most antigenically similar while BR07 and CA09 HA antigens were largely, but not completely, antigenically distinct. The antigenic-site, Ca1, of BR07 was the most antigenically similar (BR07 vs CA09: Sa = 8 AD, Sb = 15 AD, **Ca1 = 7 AD**, Ca2 = 10 AD, Cb = 13 AD) and the only antigenic-site predicted to have a close enough antigenic distance to allow an antibody to bind both antigens (AD < 8, see Methods). Alternatively, SC18 and CA09 had 4 antigenic-sites with an AD of less than 8 (SC18 vs CA09: **Sa = 2 AD**, **Sb = 3 AD**, **Ca1 = 5 AD**, Ca2 = 8 AD, **Cb = 3 AD**). Overall, in the model the HA antigens of SC18 and CA09 influenza viruses had the greatest number of antigenically similar antigenic-sites while BR07 and CA09 HA antigens were largely antigenically dissimilar.

### Evaluation of pre-existing immunity

Two models were created representing individuals with different exposure histories (Fig. 1). The first model represents younger-aged individuals in 2009 that were originally exposed to recently circulating influenza viruses (e.g. BR07) that were antigenically distinct from the 2009-pandemic (e.g. CA09) virus. The second model represents older-aged individuals that were originally exposed to early twentieth century H1N1 viruses (e.g. SC18) that contained an HA antigen that was more antigenically similar to the HA antigen of 2009-pandemic virus (e.g. CA09). B cell and antibody levels to each antigenic-site of each HA in the models were tracked throughout the simulations.

**Fig. 2:**
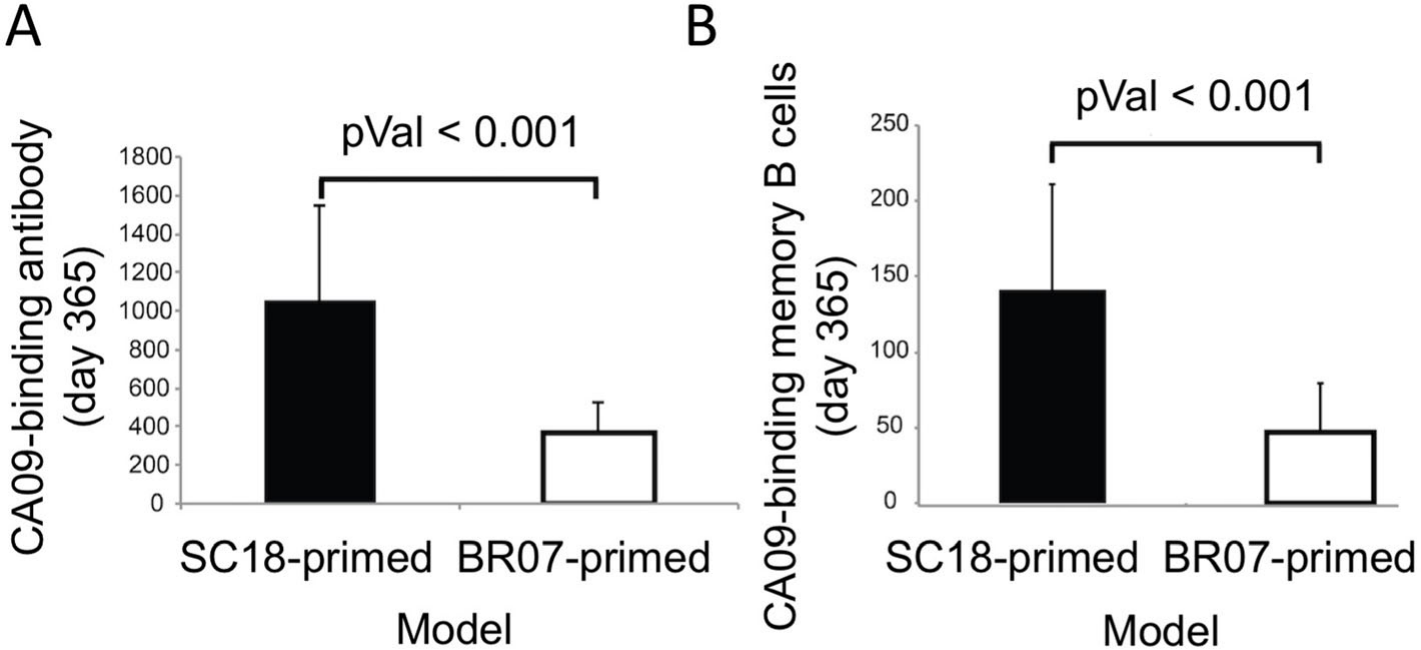
CA09-Specific Cross-Reactive Immunity Prior to Boosting. **a** The number of cross-reactive antibodies and **b** memory B cells specific to the CA09 HA antigen just prior to boost (Day 365) in each model. Error bars represent standard deviation between the simulations. Statistic represents result of two-sample t-test

The level of antibody and memory B cells cross-reactive to the HA antigen of the 2009 pandemic virus just prior (day 365) to immunization was determined for each model. As expected due to the shorter antigenic-distance between SC18 HA antigen and CA09 HA antigen, both cross-reactive antibodies and memory B cells specific to CA09 were significantly lower in the BR07-HA primed model (Model 2) compared to the SC18-HA primed model (Fig. 2a, b). Therefore, the model demonstrated the differences in the pre-existing cross-reactive immunity expected due to differences in the antigenic relationship between the priming and boosting antigens.

### Differences in antibody specificity and cross-reactivity after secondary exposure

**Fig. 3:**
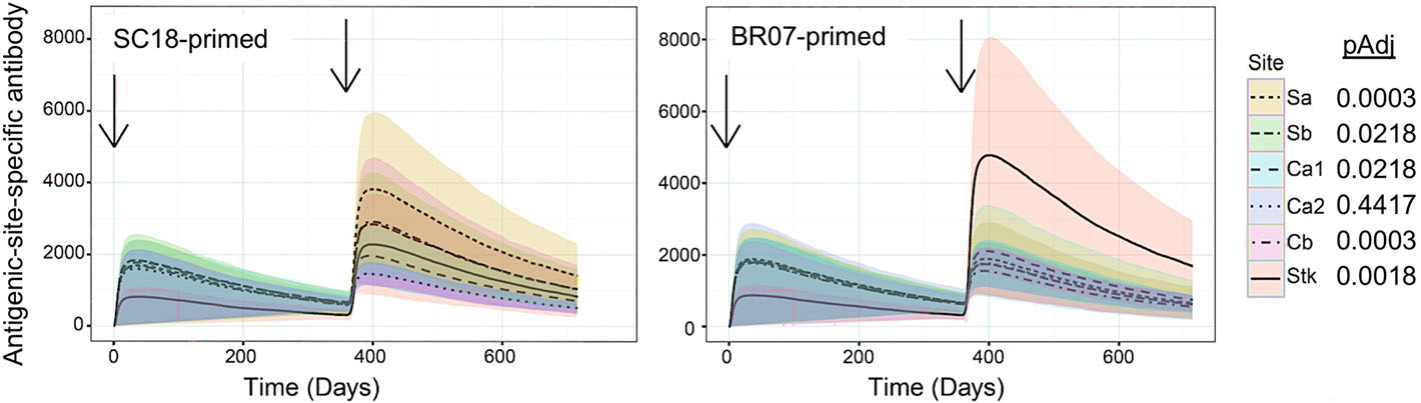
Antigenic-Site Specific Total Antibody Responses. The number of Antigenic-site-specific antibodies throughout the simulation for the SC18-primed (left) and BR07-primed (right) models. Curves represent average titers for 50 simulations and colored area represents the standard deviation. Arrows represent times simulations were primed and boosted. Values in the key are adjusted *p*-values from comparison of antibodies levels between models day 30 post-boost

The antigenic-site specificity and cross-reactivity of simulated antibody 30 days (day 395) after boosting with CA09 HA antigen was determined in the two models. After boosting, levels of antibody specific to CA09 antibody was slightly lower in the BR07-HA primed model compared to antibody in the SC18-HA primed model, although this did not reach significance (p = 0.072). Antibodies to all antigenic sites, except for the Ca2 antigenic site, were significantly different between models (Fig. 3 key). In the SC18-primed group, antibodies to the Sa-antigenic-site of the HA of CA09 influenza virus dominated, while antibodies to the HA Stk-antigenic-site dominated after boosting in the BR07-HA primed group (Fig. 3). Taken together, antigenic-site immunodominance differed between models.

**Fig. 4:**
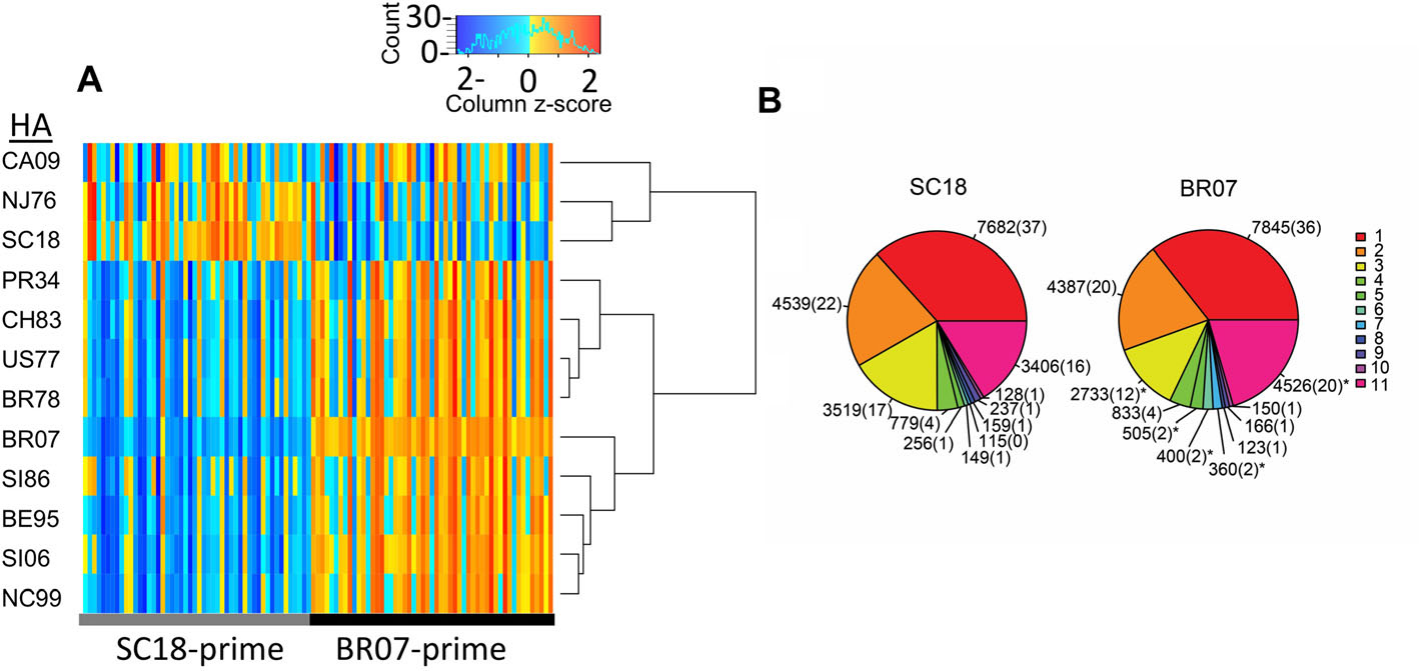
Cross-reactivity After Boosting with CA09. **a** Comparison of antibody levels to HA antigens in the SC18-primed and BR07-primed models after boosting with CA09. Each column represents an HA antigen from a single influenza strain. Each row represents a single simulation. The model is indicated by the black and white bar. Antibody levels were taken at 30 days post-boost (day 395) and log transformed. **b** For both models, the number of HA antigens (1–11) that an antibody could bind was determine for each antibody present 30 days post-boost. The pie-chart is the number of antibodies able to bind 1–11 HA antigens. The number in the parenthesis is the percentage of the total antibodies present at 30 days post-boost cross-reactive to 1–11 HA antigens

The level of cross-reactive antibody also differed between the models. Cross-reactivity was determined by counting the number of antibodies in the simulation that can bind with some affinity (AD < 8) to any antigen in the model. The level of cross-reactive antibodies 30 days post-boost to a set of 11 HA antigens representing historical/vaccine influenza viruses were compared. Both models showed strong antibody responses to the antigens to which they had been previously exposed, but differed largely in responses to the other influenza viruses. In the SC18-HA antigen primed model (Model 1), the total number of antibodies in the simulation was highest for the SC18 HA antigen and antigens antigenically similar to SC18 (i.e. CA09, NJ76). The BR07 model (Model 2) had the most cross-reactive antibodies to all other strains (Fig. 4a). Additionally, in the BR07-HA primed model showed an increase in highly-cross-reactive antibodies compared to the SC18-HA primed model (Fig. 4b). Taken together, the BR07-HA primed model, where the antigenic distances between head antigenic-sites were large, showed the greatest level of cross-reactivity.

### Comparison of simulation results with humans serum antibody

Given that most individuals are primed within the first few years of life, either by natural infection or vaccination, those born during the early twentieth century are expected to have been originally exposed to early twentieth century influenza virus strains (e.g. SC18). Conversely, those born more recently in 2009 are expected to have been originally exposed to twenty-first century viruses (e.g. BR07). Human blood serum samples taken 30 days after immunization with the 2009 monovalent H1N1 pandemic vaccine from two age-groups (18–32 years-old, 70+ years-old in 2009) was obtained. Antibody levels in the serum using a set of recombinant HA proteins from a subset of strains used in the model was measured. The simulation results from our two models with actual levels of human serum antibody was compared.

**Fig. 5:**
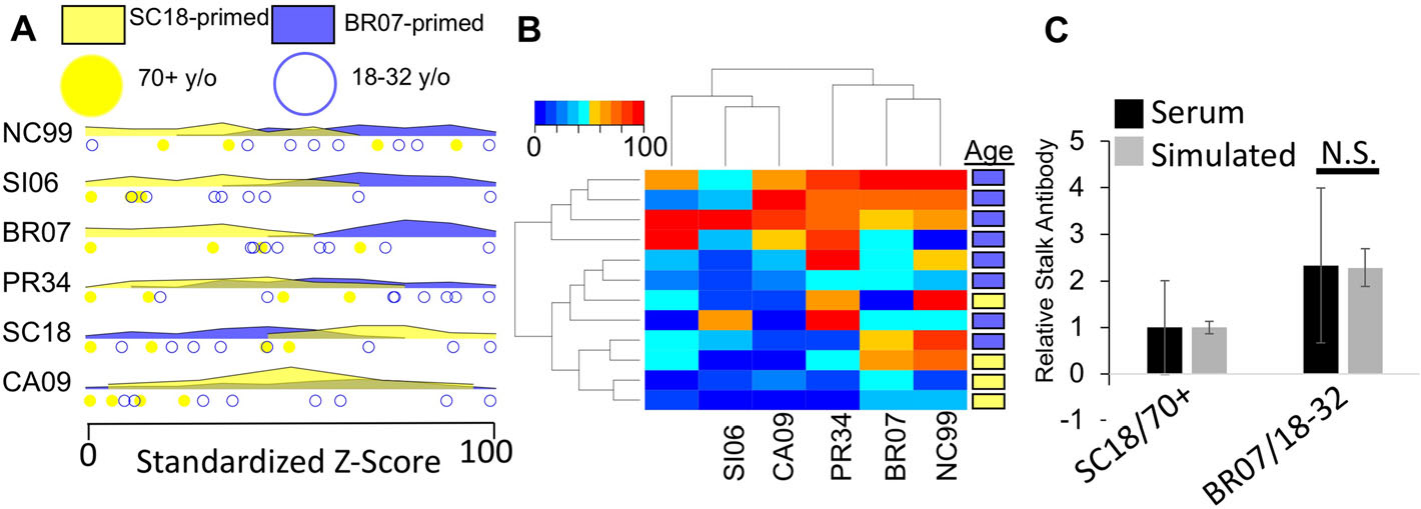
Comparison of Human Serum Antibody Levels with Simulation Data. **a** Standardized simulated antibody levels 30 days post boost are represented as ridgeplots for each HA antigen. Standardized human serum antibody binding levels for two-age groups (18–32 years-old and 70+ years old) for each recombinant HA proteins are represented by dots below the ridgeplots. **b** Heatmap of the levels of human serum antibody binding level for each recombinant HA protein for both age-groups. Blue rectangles represent serum taken from younger individuals (18–32 years-old) and yellow rectangles represent older individuals (70+ years-old). **c** Relative fold change of day 30 post-vaccination serum antibody levels specific to the stalk region of the HA protein compared to simulation antibody levels to the stalk antigenic-site taken 30 days post-boost. Error bar represents standard deviation

Human serum antibody levels after vaccination with the monovalent 2009 pandemic vaccine generally differed between age-groups, although the difference depended on the recombinant HA protein to which antibody binding was measured. Overall, 18–32 year-olds had higher antibody levels to the 6 recombinant HA proteins, similar to the BR07-HA primed model (Fig. 5a). These results were qualitatively similar to the simulation data, except for the SC18 HA antigen which was inconsistent (Fig. 5a). Hierarchical clustering of antibody binding data was generally associated with age, although some individuals clustered with individuals from the other age group (Fig. 5b). Taken together, serum antibody levels were highest in individuals expected to have been originally exposed to an HA antigen antigenically dissimilar to the 2009 pandemic vaccine HA antigen, similar to what was seen in the simulations.

Lastly, we measured the levels of antibody to the stalk region of CA09 HA antigen using a recombinant HA protein containing an exogenous head and conserved stalk domain in the two age groups. We found that antibody levels to the stalk region of HA differed between age groups (Fig. 5c). The 18–32-year-old group showed an approximate 2-fold increase in stalk antibody compared to the 70+ group, similar to the simulation results. Taken together, the age groups differed in stalk-specific-antibody levels taken 30 days after they received the 2009 pandemic vaccine in a manner similar to those seen in the simulations.

## Discussion

The 2009 H1N1 pandemic vaccine induced antibodies able to bind to antigenically distinct viruses in young adults, but not older adults, due to differences in the antigenic-site-specificity of their antibody response [3, 19, 22–24]. Here, we presented a computational model that captured many of the differences in antigenic-site-specificity, and resulting antibody cross-reactivity, seen in different age-groups after vaccination with the 2009 H1N1 pandemic vaccine.

Our results were consistent with reports suggesting that an individual’s original virus exposure affected the vaccine response to the 2009 H1N1 pandemic vaccine, including differences in pre-existing cross-reactive immunity, HA specificities, and antigenic-site dominance [12, 13]. Specifically, prior to exposure to the 2009 pandemic vaccine, older individuals were found to have increased levels of cross-reactive antibody and memory B cells to the 2009 pandemic strain [7], similar to the results of our model. Comparison of cross-reactive antibody levels between the SC18-primed model and the BR07-primed model prior to boosting with CA09 HA antigen showed an almost 3-fold greater level in the BR07-primed group, similar to what has been reported comparing young individuals to older individuals [25]. Additionally, the almost 2-fold-change increase in the antibody response to the stalk seen in the BR07-primed model is consistent with published reports on younger individuals [7]. In the simulations, the antigenic-site (Sa), which had the least antigenic difference among SC18 and CA09 HA head antigenic-sites, dominated the antibody response after boosting with CA09 in the SC18-HA primed model. The Sa antigenic-site dominance in the SC18-HA primed model is consistent with experimental data showing that antibody responses from the 60+ year old individuals had antibody responses to the Sa site of CA09 HA antigen [26]. Furthermore fold-change titers (pre-boost/post-boost) were decreased in the SC18-HA primed model suggesting that priming history, not just immunosenescence, was responsible for the difference in antibody increases seen in different age groups [27–30]. Taken together, antibody cross-reactivity and specificity in our simulations were both quantitatively and qualitatively similar to what has been reported in humans.

Lastly, our results suggest that individuals may be expected to respond differently to influenza vaccination, especially when the vaccine is antigenically distinct from recently circulating strains. The current World Health Organization (WHO) criteria for updating the vaccine strain is largely dependent on the antigenic distance between the vaccine and circulating strains [31]. Generally, if the average antigenic distance of the current vaccine strain is more that 2 antigenic distance units with circulating strains, then the strain is updated to a strain more antigenically related to the circulating strains. Although antigenic distance methods used by the WHO have been shown to be reliable indicators of the cross-reactivity expected after vaccination, our results suggest that cross-reactivity will likely depend on both antigenic distance and to which influenza antigens an individual was previously exposed.

### Assumptions & sources of errors

There are a number of assumptions in the model that should be discussed here. First, the model assumed that exactly five antigenic-sites exist on the head of the HA antigen as well as a single stalk antigenic-site. In reality, other antigenic-sites have been suggested including at least two in the stalk region [32]. Novel antigenic-sites can be easily added to the model and antigenic distances for the antigenic-site can be estimated using the previously described method [4, 33, 34]. Another assumption of the model is that affinity was modeled as a discrete variable in a manner consistent with others [23, 28], but in reality, affinity occurs on a continuous scale. Furthermore, the strict cross-reaction cutoff of seven may be incorrect, although consistent with immunological data [35], and the cutoff should be re-explored experimentally. We want to also acknowledge that the timing between antigens exposures (365 days) in our simulations was not realistic, as almost 100 years had occurred between 1918 and 2009 virus strain circulation. Lastly, the small sample size in the clinical trial makes it difficult to draw significant conclusions**.** Taken together, our model is able to reproduce many aspects of humoral immunity seen in real-life scenarios, although some of the underlying biological processes in the model are coarsely represented.

## Conclusions

In conclusion, we show that sequence-based antigenic distance measurements can be used to estimate antigenic parameters for virtual antigens in a computer model of the humoral immune system. We show that the model captures the effect previous influenza antigen exposure has on the humoral immunity, including antibody cross-reactivity and antigenic-site immunodominance. Our findings are consistent with other studies that suggest that the antigenic properties of the HA to which an individual was first exposed affects their B cell repertoire in a way that skews future antibody responses in an antigenic-site-specific manor [9, 21, 28, 29, 36, 37].

## Methods

### Sequence-based antigenic distances estimation

The antigenic distance between HA antigens was estimated using a previously described method [4, 34]. In short, the protein-coding-regions of the HA for each strain were obtained from publicly available influenza genomes. In silico translation was performed and protein sequences were aligned using the MUSCLE approach [38, 39]. For each of the 5 canonical H1N1 antigenic-sites (Sa, Sb, Ca1, Ca2, Cb), the translated linear protein sequences were truncated to only include amino acids comprising those antigenic sites [4]. Pairwise Hamming distances between truncated sequences were calculated, giving the number of amino acid differences in each antigenic-site for each influenza strain. Hamming distances were then divided by the number of amino acids in each antigenic-site, resulting in the percentage of difference. This number is multiplied by 20, resulting in an antigenic distance estimate for each antigenic site in a 20-character shape space [35].

### Immunological shape space computational model

**Fig. 6:**
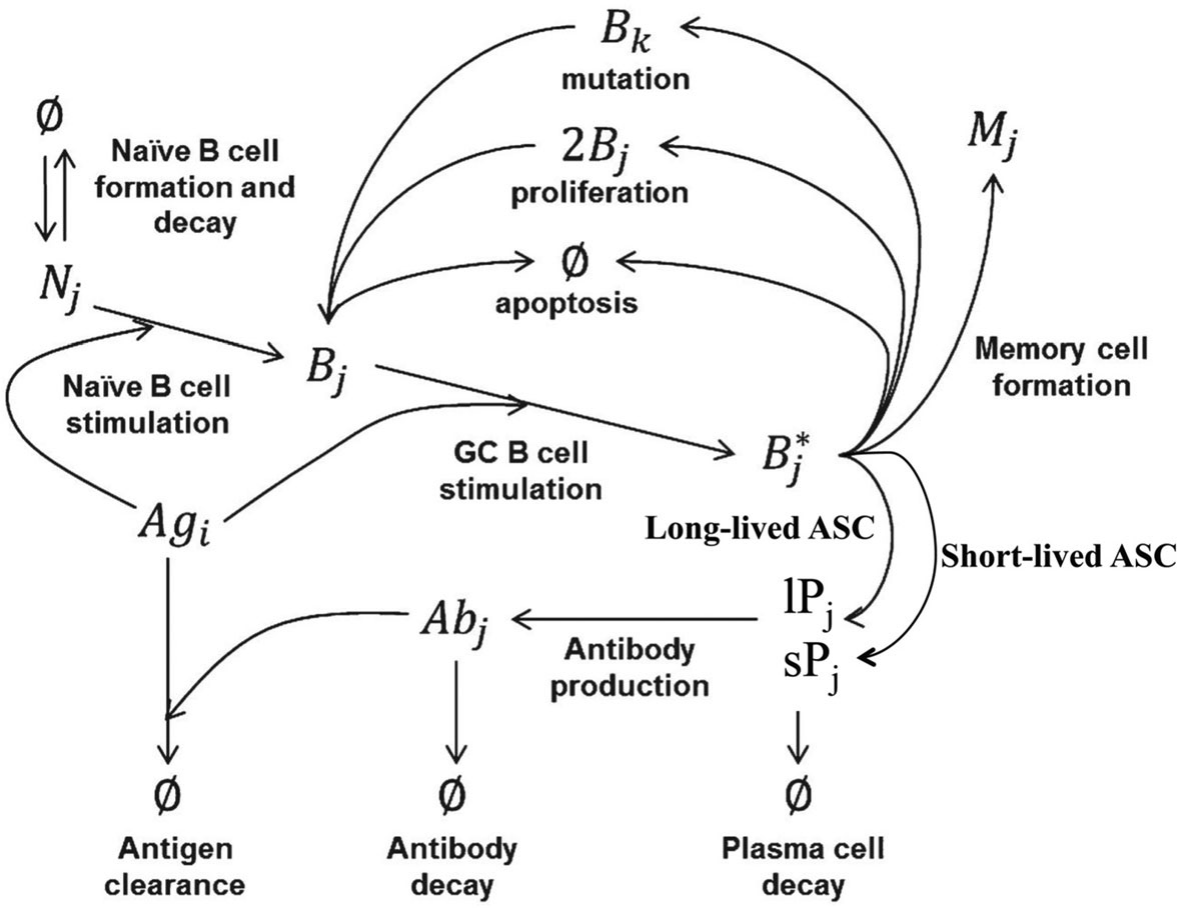
Schematic of Immunological Shape Space Model. The computer model is comprised of a set of agents (B cells, antibody, and antigen). Biological processes are governed by a set of rate equations. Simulations are performed using a master equation (Gillespie algorithm) which executes rate equations based on their probability of occurring. Lines represent interactions between agents and processes. Schematic is revised from Chaudhury et al. 2014 [23]

The model developed by Chaudhury et al. 2014 [23] was used for this study except for two modifications: (1) the number of antigenic-sites representing each antigen in the model was increased from 2 to 6 (2) long-lived plasma cells were added to the model using previously published parameters [28] (Fig. 6). The model represents an artificial humoral immune system sensitive to antigenic changes in virus antigens [28, 40].

In brief, an antigenic-site was represented in the model as a 20-character string. The strings are created in such a way that the number of characters different between the strings was equal to the antigenic distances between HA antigenic-sites they represented. For this study, antigens in the model contained 6 antigenic-sites, representing the five canonical head antigenic-sites on the HA protein and a fully conserved stalk antigenic site (AD = 0, [32]). A 4-letter alphabet 20-character shape space provides the characteristics needed to represent antigen-immunoglobulin interactions. This space allows ~1X10^12^ unique characters (shapes). Parameters for a such a shape space have been previously derived and demonstrated that a genetic change of 30–40% between two antigens results in loss of cross-reactivity between antigens [35, 41]. Therefore, antigenic-sites with an antigenic distances of 7 or less (cross-reactive cutoff) were considered to be close enough in shape space for antibodies in that space to cross-react with both antigens in the model [23, 28].

The model simulates a simplified humoral immune system response to exogenous antigen. The agent-based simulation begins by the creation of “naïve” B cells. Each naive B cell contains an immunoglobulin receptor represented as a 20-character, 4-alphabet, string (e.g. “AAAAABBBBBCCCCCDDDDD”). Immunoglobulin strings are generated by a random number generator using a computationally efficient method previously described [42]. Naive B cells are continually generated and naturally decay. Upon exposure to antigen, naïve B cells become stimulated, differentiate into memory B cells and plasma cells. Plasma cell then secrete their immunoglobulin (antibody) which is able to bind the antigen and remove it from the system. Memory B cells become activated and differentiate at a faster rate compared to naïve B cells during secondary exposure to similar antigens.

#### Software

The source code for the ssMod.v1 Version 1 is available at the GitHub repository, https://github.com/canderson84/ssMod.v1. It is implemented in Python 3.

### Models representing 2009 pandemic vaccination

Two scenarios were modeled using the immunological shape space computational model described above. These models represent those vaccinated with the 2009 H1N1 pandemic vaccine who had been exposed to HA antigens from 1918-pandmeic-like strains or HA antigens from more recent strains. Specifically, in one modeled scenario (SC18-primed) a simulation occurred were the model was primed with the 1918-pandemic strain, A/South Carolina/01/1918 (SC18), HA antigen and 1 year later was boosted with the 2009-panemic strain, A/California/07/2009 (CA09), HA antigen. The second modeled scenario (BR07-primed) was identical to the first except priming was done with the 2008–2009 vaccine strain, A/Brisbane/59/2007 (BR07), HA antigen. The number of simulations was varied to determine after how many replications the results converge; 50 simulations were chosen (Supplemental Figure). Memory B cells and antibodies, including counts, genotype, and antigen specificities were tracked throughout the simulation.

### Influenza strains represented in the model

Influenza strains were chosen based on historical significance (pandemic and vaccine strains). Additionally, strains were chosen such that the ADs from BR07 to the 11 strains was not significantly different from the ADs from SC18 to the 11 strains (two-sample t-test, *p*-value = 0.362). Influenza HA genome sequences used in the model were obtained from the Influenza Resource Database (fludb.org): A/California/07/2009 (CA09) [NC_026433], A/Brisbane/59/2007 (BR07) [KP458398], A/South Carolina/01/1918 (SC18) [AF117241], A/Beijing/262/1995 (BE95) [AAP34323], A/Brazil/11/1978 (BR78) [A4GBX7], A/Chile/1/1983 (CH83) [A4GCH5], A/New Caledonia/20/99 (NC99) [AY289929], A/Singapore/6/1986 (SI86) [ABO38395], A/Solomon Islands/3/2006 (SI06) [ABU99109], A/USSR/90/1977 (US77) [P03453], A/New Jersey/11/1976 (NJ76) [ACU80014], A/Puerto Rico/8/1934 (PR34) [HQ008261].

### Human serum antibody binding after 2009 H1N1 monovalent vaccination

The previously reported clinical trial was conducted under a protocol approved by the University of Rochester Research Subjects Review Board [7]. Informed written consent was obtained from each participant or parent/guardian for minors. ClinicalTrials.gov identifier NCT01055184. Healthy adults and children were enrolled as previously described and results of this clinical trial have been published previously [7]. Subjects received a single intramuscular (i.m.) injection of inactivated influenza A/California/07/2009 (H1N1) monovalent subunit vaccine (Novartis). Each 0.5-ml dose contained 15 μg of HA antigen. Administration of the vaccine (study day 0) took place from January 2010 to March 2010. Serum was collected before and 28 days after vaccination. A subset of the deidentified residual serum samples from this study was used including 8 samples from those 18–32 years old and 4 samples from those 70+ were used for this study.

Serum antibody binding was determined by enzyme-linked Immunosorbent Assay (ELISA). Serum IgG HA-specific antibody levels were measured using recombinant HA proteins by indirect-ELISA. Serum antibody levels to 6 recombinant HA proteins [Influenza Reagent Resource: Cat#: FR-67 (SI06-rHA), FR-692 (SC18-rHA), FR-65 (BR07-rHA), FR-180 (CA09-rHA) and BEI Resources (Cat# NR-19240 (PR34-rHA)]. Stalk antibody levels where determined using a chimeric recombinant protein containing the H1N1 stalk region and an exogenous (H9/H1-rHA) head region. Recombinant HA protein was coated on MaxiSorb 96-well plates (ThermoSci; 439,454) overnight at 4 °C. Plates were blocked with 3% bovine serum albumin (BSA) in phosphate buffered saline (PBS) for 1 h at room temperature. Serum was diluted 1:1000 in PBS/0.5% BSA/0.05% Tween-20. Plates were washed and incubated with alkaline phosphatase (AP)-conjugated secondary antibody for 2 h at room temperature. Plates were washed and developed using AP substrate (ThermoSci 34,064). Fold-change (d28/d0) antibody levels for each HA were calculated. Z-scores were calculated and data was scaled from 0 to 100 for each HA for both simulation and ELISA data. Histograms (R base packge) of simulations data was used for ridgeplots for comparison to human data.

### Statistics

Two sample, two-tailed, t-test using the *t.test* function was performed using the base packages in *R 3.4.4*. A *p*-value of 0.05 or less was considered statistically significant. Multiple correction testing was performed using the Benjamini-Hochberg correction method in *R 3.4.4*.

## Supplementary information

**Additional file 1.**

## Data Availability

Antigenic distances and codes for the models used to produce the data in these studies are available as supplemental data.

## Abbreviations

AD: Antigenic distance
SC18: A/South Carolina/1/1918 influenza pandemic strain
BR07: A/Brisbane/59/2007 influenza vaccine strain
CA09: A/California/07/2009 influenza pandemic vaccine strain
ELISA: Enzyme-linked Immunosorbent Assay
