# Supplementary material for: Implementing sequence-based antigenic distance calculation into immunological shape space model
Source: BMC Bioinformatics. 2020 Jun 19;21:256. doi: 10.1186/s12859-020-03594-3 (PMC7303933; doi:10.1186/s12859-020-03594-3)
Supplement: Supplementary file 1 — Additional file 1. [file 12859_2020_3594_MOESM1_ESM.zip › SupDocs/Supplemental_Fiigure.pdf]

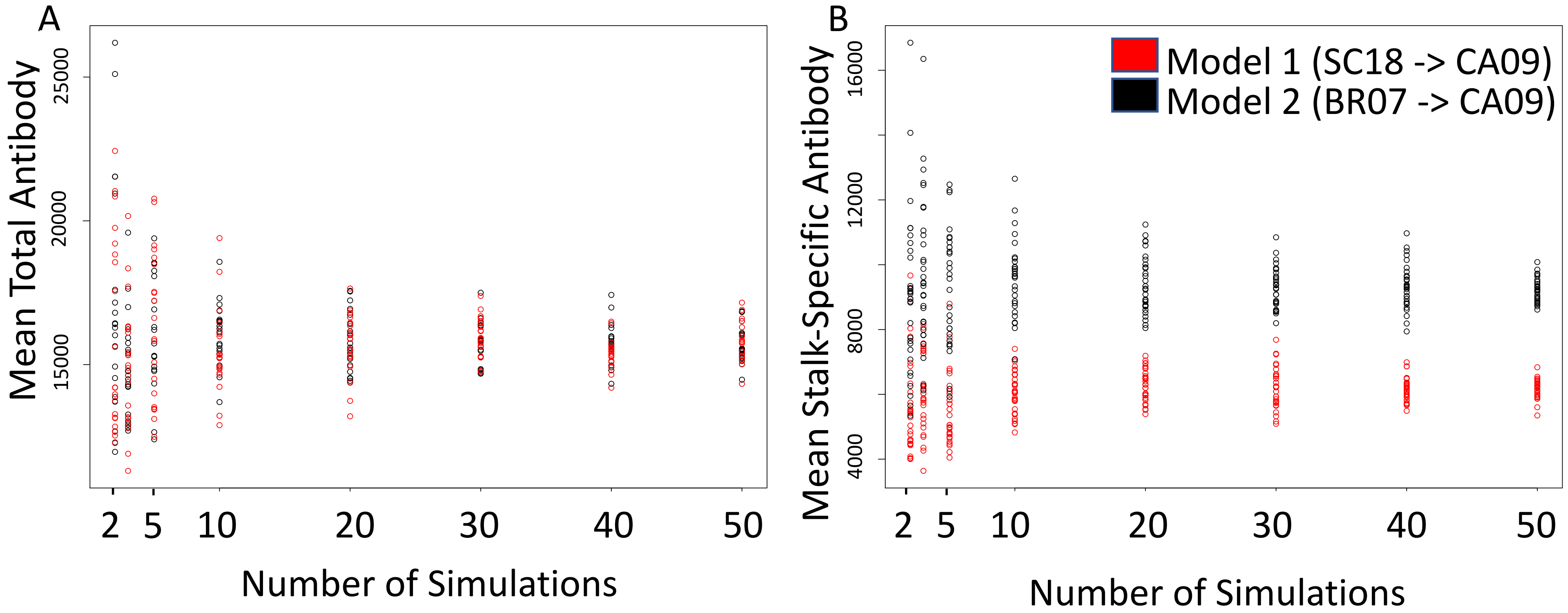

Effect of the number of simulations on mean value of the simulations. Each model was simulated 2– 50 times and the mean number of total antibodies or antibodies specific to the stalk-antigenic-site at day 30 post-boost with CA09 HA antigen was calculated. This process was repeated 30 times. (A) Each dot represents the mean total antibody level obtained for each repeat for the specified number of simulations for each model. (B) Each dot represents the mean stalk-specific antibody level obtained for each repeat for the specified number of simulations for each model.
